# Therapeutic Vaccines and Antibodies for Treatment of Orthopoxvirus Infections

**DOI:** 10.3390/v2102381

**Published:** 2010-10-20

**Authors:** Yuhong Xiao, Stuart N. Isaacs

**Affiliations:** 1 Division of Infectious Diseases, University of Pennsylvania, School of Medicine, Philadelphia, PA 19104, USA; E-Mail: yuhong2@mail.med.upenn.edu; 2 Infectious Diseases Section, Philadelphia Veterans Affairs Medical Center, Philadelphia, PA 19104, USA

**Keywords:** orthopoxvirus, variola virus, vaccinia virus, monkeypox virus, post-exposure vaccination, Dryvax, Lister, modified vaccinia Ankara (MVA), vaccinia immune globulin (VIG), monoclonal antibodies

## Abstract

Despite the eradication of smallpox several decades ago, variola and monkeypox viruses still have the potential to become significant threats to public health. The current licensed live vaccinia virus-based smallpox vaccine is extremely effective as a prophylactic vaccine to prevent orthopoxvirus infections, but because of safety issues, it is no longer given as a routine vaccine to the general population. In the event of serious human orthopoxvirus infections, it is important to have treatments available for individual patients as well as their close contacts. The smallpox vaccine and vaccinia immune globulin (VIG) were used in the past as therapeutics for patients exposed to smallpox. VIG was also used in patients who were at high risk of developing complications from smallpox vaccination. Thus post-exposure vaccination and VIG treatments may again become important therapeutic modalities. This paper summarizes some of the historic use of the smallpox vaccine and immunoglobulins in the post-exposure setting in humans and reviews in detail the newer animal studies that address the use of therapeutic vaccines and immunoglobulins in orthopoxvirus infections as well as the development of new therapeutic monoclonal antibodies.

## 1. Introduction

Orthopoxviridae is a genus of poxviruses that includes viruses such as variola virus (VARV), the causative agent of smallpox, vaccinia virus (VACV), the vaccine used to help eradicate smallpox, and cowpox virus and monkeypox virus (MPXV), viruses that cause zoonotic infections of humans. These are large DNA viruses that encode hundreds of proteins that induce strong, cross-reactive immune responses. Thus, infection by one of these viruses results in protection from disease caused by another.

Despite the successful worldwide eradication of smallpox by the World Health Organization [1], VARV and MPXV have the potential to become serious public health threats [2,3]. Live VACV-based vaccines were extremely effective in generating remarkably long-lived immune responses [4–6] and protection from VARV and MPXV [1]. However, due to serious but rare vaccine complications [7], routine smallpox vaccination ceased decades ago. Thus, in the potential setting of new human infections with poxviruses, post-exposure smallpox vaccination and/or immunoglobulins may become first line therapies. Post-exposure vaccines and immunoglobulins have been successfully used against smallpox and other viral infections like those caused by hepatitis A and B viruses, rabies virus, and varicella zoster virus [8]. In this article we will discuss some of the old human studies and more extensively review the newer animal studies of the therapeutic use of live VACV vaccines in the post-poxvirus exposure setting and the use of immunoglobulins for therapy against poxvirus infections.

## 2. Post-exposure Vaccination

### 2.1. Studies of Post-exposure Vaccination of Humans with Conventional Replication Competent Smallpox Vaccines like Dryvax® or Lister

While prophylactic vaccination with live VACV vaccines helped eradicate smallpox from plaguing humankind, if given after exposure, the vaccine could modify the progress of smallpox disease or even prevent it if given within the first few days after exposure. Based on the available information at the time, Dixon concluded in his book “Smallpox” [9] published in 1962, that “… I think it is probably fair to say that at least 50 per cent of cases where successful vaccination has occurred in the first week will get some [vaccine] modification and reduction of severity, whereas when done at a later period the number showing such modification is not likely to be over 20 per cent.”

An old study by Dr. William Hanna, which was recently republished [10], was an early example of a report that showed the effect of post-exposure vaccination. Hanna collected smallpox cases presenting to a hospital in Liverpool, UK during the early 1900s and found that if a primary or boost vaccination was given during the incubation period (*i.e.* before onset of symptoms), smallpox disease could be altered. Because the cases he collected had smallpox, the data did not include cases that might have been fully protected by post-exposure vaccination. He reported on 19 cases exposed to smallpox that had previously never been vaccinated and were vaccinated during the incubation period. He found that two (10.5%) developed no symptoms, eight (42%) showed mild symptoms, seven (37%) showed moderate symptoms, and the vaccine did not alter the disease in two (10%) who developed severe symptoms. In contrast to vaccination before the onset of symptoms, he reported on 11 cases (previously never vaccinated) that were given vaccine after the onset of symptoms. These symptomatic patients did not develop the characteristic vesiculopustular lesions following vaccination and three (27%) died, five (46%) developed severe disease, and three (27%) showed moderate disease. The reasons for not developing characteristic vaccine-related skin lesions could have been due to a failure of the vaccination procedure, or in these individuals, who had never been vaccinated, it is possible that the immune response to the VARV infection might have suppressed VACV replication (similar to individuals who have anti-vaccinia immunity from very recent or multiple vaccinations). Post-exposure vaccination was more successful in those previously vaccinated. In the groups that had prior vaccination, he reported on 25 cases revaccinated during the incubation period and found that 21 (84%) showed mild symptoms, three (12%) showed moderate symptoms, and the vaccine did not alter disease in one (4%) who developed severe symptoms. In the 19 cases revaccinated after development of symptoms, the formation of vesiculopustular lesions after vaccination were poor (perhaps again due to development of early immune responses to VARV), and in this group nine (47%) showed mild symptoms and 10 (53%) showed moderate symptoms, likely indicating some level of protection from their past primary vaccination [10].

A similar modification in disease by post-exposure vaccination was suggested by Rao [11]. He found that among those given primary vaccination at the time of exposure, 44 of 502 exposed (8.8%) developed modified-type smallpox, while only 15 of 1,453 exposed (1.0%) among unvaccinated patients developed modified-type smallpox. However, both groups developed ordinary type smallpox at similar rates: 85% (426 of 502) in the vaccinated group and 89% (1296 of 1453) in the unvaccinated group. Using slightly different outcome measures, Heiner *et al.* showed a greater effect of post-exposure vaccination [12]. In a group of 53 people who were vaccinated within seven days after smallpox exposure, only one (1.9%) developed smallpox. While in a group of 412 people exposed to smallpox who did not get vaccinated, 90 (21.8%) developed smallpox. However, in this study, it was not clear how many of these smallpox exposed people had been previously vaccinated against smallpox. Within a larger epidemiologic study of smallpox outbreaks in Pakistan, Mack *et al.* showed some effect of post-exposure vaccination in previously smallpox vaccine naïve patients [13]. They identified 43 people who were never vaccinated and exposed to an active case of smallpox. Of those who did not get vaccinated within 10 days of the exposure, 26 of 27 (96%) developed smallpox. In those who received vaccination within 10 days of the exposure, 12 of 16 (75%) developed smallpox. Based on these types of observational studies, which are the only available data on post-exposure vaccination, it is generally accepted, that if given early enough after exposure, post-exposure vaccination with live VACV vaccines can modify and potentially prevent smallpox disease. That is, primary vaccination done early after exposure could at least protect individuals from serious illness and revaccination during the first week of exposure of previously vaccinated individuals could prevent smallpox. Such conclusions have been reached in a recent analysis of old data sets [14].

### 2.2. Animal Studies of Post-exposure Vaccination with Replication Competent VACV Vaccines

Since the eradication of smallpox, all data on post-exposure vaccinations for the treatment of poxvirus infections have been generated using animal models. In these studies, post-exposure vaccination supports the notion that vaccination may provide an effective means to decrease the morbidity and mortality from poxvirus exposures in the emergency setting. However, an important caveat to all of these studies is that challenge doses and challenge routes do not recapitulate the natural acquisition of VARV and MPXV infections. That is, the challenge doses are likely much higher than what may normally initiate a natural infection and the routes of infection do not authentically mimic natural infection.

Initial studies with replication-competent VACV vaccine strains did not show protection. For example, Staib *et al.* found no survival when VACV Elstree was given just prior to or after intranasal challenge with VACV [15]. In this study, mice were vaccinated with 1 × 10^6^ pfu (plaque forming units) VACV-Elstree by scarification 2 days prior to challenge or on day 0, 1, or 2 after intranasal challenge with 1 × 10^6^ pfu VACV (strain WR). While vaccination with Elstree at times around challenge failed to confer protection against this high dose intranasal challenge, mice vaccinated 14 days prior to challenge were fully protected. Similar failure of post-challenge vaccination was seen with an intratracheal challenge of nonhuman primates with MPXV [16]. Only one of six macaques survived a lethal intratracheal challenge with 1 × 10^7^ pfu MPXV if vaccinated with VACV Lister/Elstree strain (2.5 × 10^5^ TCID_50_) by scarification 24 hours after challenge. The other five macaques met end point criteria at ∼10 days after challenge.

However, successful vaccination near challenge could be seen in other types of animal models. Paran *et al.* studied post-exposure vaccination with VACV-Lister against lethal ectromelia (mousepox) virus (ECTV) infection [17]. Balb/c mice, which are highly susceptible to ECTV infection, were vaccinated with 10^6^ pfu VACV-Lister by intradermal or intramuscular routes on the day they were intranasally challenged with ECTV (3 × LD_50_). They found that vaccination, by either route, on the day of challenge conferred 100% protection and that vaccination, by either route, when given one day after challenge resulted in 80% survival. However, when vaccination was given two days after challenge, the route of vaccination appeared to slightly impact on survival. They found only a 16% survival rate after intradermal vaccination and 40% survival rate after intramuscular post-challenge vaccination. They noted that the mean time to death of ECTV infection in Balb/c mice is ∼10 days, while for intranasal VACV-WR challenge, death occurs in ∼6 days. Thus, as opposed to mice intranasally challenged with WR [15] or monkeys intratracheally challenged with MPXV [16], the protection seen with the VACV-Lister vaccine in ECTV challenged mice could be due to the differences the mean time to disease. When compared to VACV challenge, ECTV infection appeared to have a longer lag period before the onset of morbidity and mortality. ECTV has a ∼7 day incubation period, while VACV has only a 2–3 day lag period before the onset of symptoms. The longer time for ECTV to cause disease may allow the replication competent vaccine to induce an initial adaptive immune response that results in protection. For VACV or MPXV pneumonia models, disease is faster and apparently outpaces the time needed for an initial immune response. Indeed, robust protection of mice against intranasal VACV challenge could be generated if vaccine was given 14 days prior to challenge [15]. Since natural infection with VARV has an incubation period of ∼12 days, the ECTV challenge-vaccination models support the earlier observations that humans vaccinated at early times after VARV exposure could result in less severe disease and even full protection from disease.

### 2.3. Animal Studies of Post-exposure Vaccination with Replication Incompetent VACV Vaccines like Modified Vaccinia Ankara (MVA)

Prior to the complete eradication of smallpox, it became clear that there was a need for safer live VACV vaccines. This led to the development of more attenuated vaccines like the modified vaccinia Ankara (MVA) (reviewed in [18]). MVA was isolated after extended serial passages in chick embryo fibroblast cells and the resulting virus, which lost >10% of its genome, no longer produces progeny virus in most mammalian cells. In the 1970s, MVA was tested in Germany in over 100,000 people as a pre-vaccine to decrease complications from primary vaccination with conventional smallpox vaccine. Given current concerns about bioterrorism with VARV and ongoing outbreaks of MPXV, MVA has become a lead candidate as a next generation prophylactic smallpox vaccine [18]. The efficacy of MVA in post-exposure settings has recently been studied in animal models. Interestingly, these animal studies indicate that MVA might provide more rapid protection against lethal poxvirus infections than the fully replication competent smallpox vaccines like Dryvax and Lister when standard doses of the vaccines are given at times right before or after challenge. This may be due to the fact that MVA no longer encodes many proteins involved in evading the host response to infection and thus MVA vaccination just prior to or after challenge might result in enhanced innate and/or more rapid adaptive immune responses. Also, as discussed below, the improved protection conferred by MVA may also be due to the significantly higher standard dose of MVA. Investigations into a better understanding of the mechanism by which MVA protects in the therapeutic setting are ongoing.

This rapid protection by MVA was first shown in mice [15]. As opposed to no survival of mice vaccinated with the replication competent VACV-Elstree just prior to or after intranasal challenge with 5 × 10^4^ pfu of VACV-strain WR, mice intramuscularly vaccinated with 10^8^ pfu of MVA were protected if the MVA vaccine was given two days prior to intranasal challenge with VACV-WR. Significant protection was also seen if the MVA vaccine was given within three hours of challenge. However, similar to Elstree vaccination, MVA vaccination failed to protect if given one to four days after challenge [15].

When compared to Dryvax, more rapid protection by MVA was also seen in a MPXV-challenge of nonhuman primates when vaccines were given four days prior to challenge [19]. In this study, macaques were intramuscularly vaccinated with 1 × 10^8^ pfu of MVA or intradermally vaccinated with 2.5 × 10^5^ pfu of Dryvax at 4, 6, or 10 days prior to intravenous challenge with 5 × 10^6^ pfu MPXV. When macaques were vaccinated 10 days prior to challenge, both the Dryvax and MVA vaccinated groups survived, but there was evidence that the Dryvax group did slightly better. Animals in this group had no viremia or lesions. Two of four macaques in the MVA vaccinated group developed viremia, three developed low numbers of pock lesions, and one had no lesions. When macaques were vaccinated six days prior to challenge, the Dryvax and MVA groups had comparable protection. Two of four macaques in each group had detectable viremia and all macaques developed skin lesions. One macaque in MVA group was sacrificed on day 9 post-challenge because of high number of lesions (although its viremia was lower (∼1 × 10^5^ pfu/mL) than unvaccinated macaques (∼1 × 10^7^ pfu/mL)). When macaques were vaccinated four days prior to challenge, the MVA vaccinated group were able to control the infection and survive, while the monkeys in the Dryvax group developed viremia and lesion counts similar to the unvaccinated control group and three of four in the Dryvax group met end point criteria and were sacrificed by day 10–14. To begin to understand the difference in protection conferred by the two vaccines, immune responses were evaluated. A single injection of MVA (10^8^ pfu) induced a more rapid anti-VACV antibody response than Dryvax (10^5^ pfu). MVA also induced a more rapid CD8 T cell IFN-γ response than Dryvax. Based on these findings, the authors concluded that the more rapid immune response by MVA compared to Dryvax could be due to the fact that MVA is given at a dose that is ∼1000-times greater than the Dryvax dose. This resulted in a more rapid immune response that improved protection when MVA was given four days prior to challenge. It should also be pointed out that when the vaccines were compared in a more prophylactic setting (*i.e.* a single vaccination given 10 or 30 days prior to challenge), and with a 10-fold higher MPXV intravenous challenge dose (5 × 10^7^ pfu), the authors found that while both groups survived, the Dryvax vaccinated group was better compared to the MVA-vaccinated group based on post-challenge viral loads and skin lesion counts.

Similar to the above studies, Paran *et al.* found better protection of mice with MVA vaccination when compared to vaccination with a replication competent VACV vaccine [17]. Balb/c mice intranasally challenged with ECTV (3 × LD_50_) were fully protected when intramuscularly vaccinated with MVA (10^8^ pfu) 1 or 2 days after challenge. When vaccinations were done at 3 or 4 days after ECTV challenge, survival rates were 60% and 20%, respectively. When mice were vaccinated intradermally with VACV-Lister (10^6^ pfu) 1, 2, 3 or 4 days after challenge, survival rates were only 83%, 16%, 0% and 0%, respectively. They also found that similar to intradermal vaccination, intramuscular vaccination with VACV-Lister (10^6^ pfu) at 1, 2, 3 or 4 days after intranasal ECTV challenge gave similar survival rates of 80%, 40%, 20% and 20%, respectively. Because the MVA vaccine dose was 100-times higher than the VACV-Lister vaccine dose, they compared the vaccines given at the same dose (10^6^ or 10^8^ pfu) by the same intramuscular route of administration in Balb/c mice. With the higher vaccine dose (10^8^ pfu) given 1, 2, or 3 days after challenge with ECTV (5 × LD_50_), similar levels of protection were seen. Thus, post-challenge vaccinations with the higher vaccine doses (10^8^ pfu) showed that VACV-Lister could perform as well as MVA. But with the lower dose vaccine (10^6^ pfu) given 1, 2, or 3 days after challenge, the MVA vaccination conferred 100%, 80% and 60% survival rates, respectively, while the VACV-Lister vaccination conferred survival rates of 100%, 40% and 40%, respectively. Thus two days after challenge, MVA vaccination with 10^6^ pfu had a statistically significant higher survival rate than VACV-Lister vaccination. Thus, these authors showed that both VACV-Lister and MVA vaccination could confer post-exposure protection against lethal dose of orthopoxvirus infection, but the level of protection was associated with the vaccine dose. To begin to understand a reason for the differences in protection, they measured antibody responses at various times after MVA and VACV-Lister vaccinations (in unchallenged mice). As one would expect, they found that intramuscular vaccination with 1 × 10^8^ pfu of either VACV-Lister or MVA induced higher IgM and IgG antibodies than vaccinations with the lower 1 × 10^6^ pfu dose. Thus the difference in protection seen when Lister was given at 10^6^ pfu and MVA was given at 10^8^ could be due to the more rapid antibody response when high dose vaccines are given. To further investigate a mechanism behind short term protection by the vaccines, they examined intranasal MVA vaccination of type 1 interferon receptor deficient (IFNAR-/-) and recombination activating gene-1 deficient (RAG-1 -/-) mice. The authors focused on this route of vaccination because it appeared highly efficient and a non-invasive route of vaccine delivery. In these experiments, they intranasally vaccinated mice with MVA (10^8^ pfu) two days before intranasal challenge with ECTV (3 × LD_50_). They observed that the IFNAR-/- mice were protected with just a small amount of weight loss. However, the RAG-1-/- mice, which lack mature B and T cells, did not survive challenge. Based on these results the authors concluded that type 1 interferon is not essential for MVA-induced protection, but adaptive immune responses are needed for the short-term protection generated by MVA.

Another study that provided insight into how therapeutic MVA vaccination is protecting mice used normal and specific knockout mice [20]. They found that TLR-9 deficient (TLR-9-/-) mice were much more susceptible to intranasal ECTV challenge than the control C57BL/6 mice. However, these same TLR-9-/- mice were protected if at the time of high dose ECTV (10^4^ TCID_50_; ∼500 LD_50_) challenge they were simultaneously intranasally vaccinated with 1 × 10^8^ TCID_50_ MVA. They also investigated the efficacy of MVA vaccination in TLR-9-/- mice at times after challenge with a lower challenge dose of ECTV (10^2^ TCID_50_; ∼5LD_50_) and found full protection of mice intranasally vaccinated with MVA at 24 or 48 hours after intranasal challenge with ECTV. However, protection was reduced to ∼30% survival if mice were vaccinated 72 hours after infection. Similar to the increased susceptibility of TLR-9-/- mice to ECTV challenge, type 1 interferon receptor deficient (IFNR-/-) mice also had increased susceptibility to intranasal ECTV challenge. Unvaccinated IFNR-/- mice all succumb to challenge at 10 days. However immediate intranasal MVA (1 × 10^8^ TCID_50_) vaccination at the time of challenge protected mice challenged with 10^2^ and 10^3^ TCID_50_ ECTV, but only provided 50% and 0% survival rates for 10^4^ and 10^5^ TCID_50_ ECTV challenges, respectively. Thus these authors also found that MVA-mediated protection is partially independent of type 1 IFN responses. To examine the contribution of adaptive immune responses, the authors infected and vaccinated RAG -/- mice and found that immediate intranasal MVA vaccination after ECTV challenge could not protect, but extended the survival for several days. This data again indicates the need for the adaptive immune response to protect mice with therapeutic MVA vaccine.

This same group extended their studies to show that NK cells, neutralizing antibodies, and T cells were needed to protect mice against lethal ECTV infection after post-challenge MVA vaccination [21]. In these experiments, C57BL/6 mice were intranasally infected with a lethal dose of ECTV (3 × 10^4^ pfu) and then vaccinated with MVA (5 × 10^7^ pfu) either by intranasal or intravenous routes. Intravenous vaccination with MVA on day 2, 3, or 5 post-challenge conferred a survival rate of 100%, 100%, and 20%, respectively. Intranasal MVA vaccination at the same time points resulted in survival rates of only 30%, 0%, and 0%. To further understand the mechanism of protection conferred by post-exposure MVA vaccination, they intranasally challenged and then three days later intravenously vaccinated various transgenic mouse strains. They saw that 9 of 11 post-challenge vaccinated mice lacking NK and NKT cells (IL-15Ra deficient mice) died (most within 10 days). Similarly, in B-cell receptor transgenic mice (T11μMT mice), which are unable to produce specific anti-poxvirus antibodies, 5 of 6 mice did not survive. These results show the importance of NK cells and B-cells in post-exposure MVA vaccination. Interestingly, in mice that are unable to class switch from IgM to IgG (AID mice), MVA vaccination protected, with development of anti-pox neutralizing IgM antibodies. Similar MVA protection was found with challenged mice that lack the FcRγ chain (which have defects in all Fc-receptors and FcR signaling). When challenge-vaccination studies were done in MHC class I (β2M-/-) or class II (H2-Ab1-/-) deficient mice, they observed that 15 of 19 post-challenge MVA vaccinated MHC class II deficient mice died within 10 days, while the MHC class I deficient mice survived for three weeks and then only 3 of 12 mice survived beyond 30 days. This data indicates that CD4 and CD8 T cells are likely required for protection at different time points after post-exposure MVA vaccination. Based on these results, the authors concluded that NK cells, production of specific neutralizing antibodies, CD4, and CD8 T cells are essential for protection after post-exposure MVA vaccination. Furthermore, these conclusions indicate that the host responses required for survival after post-exposure vaccination differs from those involved in prophylactic vaccination. In prophylactic vaccination, antibodies and B cell memory alone can provide protection against high dose ECTV challenge [22–24]. But protection with post-exposure vaccination requires the presence of multiple arms of the immune system [21].

### 2.4. Conclusions

In conclusion, the animal studies reveal that immediate pre-exposure and post-exposure vaccination can protect from lethal challenge, but critical factors such as the lag period before disease of the infecting virus and the dose and route of the VACV vaccines impacts on the level of protection. It is also important to mention again that these animal models do not mimic natural VARV infection of humans. After exposure in humans, the virus replicated initially in lymphoid tissues, then disseminated through the bloodstream, perhaps making it more vulnerable to a humoral immune response. By contrast, in the animal models that use intranasal virus challenge, the presence of a localized respiratory tract infection could bring about the need for a cellular immune response to contribute to protection. The animal work with MVA is also quite encouraging since this highly attenuated live virus vaccine may actually be a better post-exposure vaccine than the conventional fully replication competent VACV vaccines. It is intriguing to consider that the improved protection conferred by MVA may depend on its loss of genes encoding immunomodulatory proteins, permitting enhanced innate immune responses and/or the more rapid development of an adaptive immune response. Also contributing to the enhanced protection is the higher immunogenic load with standard MVA vaccination, which is given at 100- to 1000-fold higher doses than the conventional, replication competent vaccines. Based on the old observational human studies done prior to the eradication of smallpox, it is likely that the fully replication competent vaccines could confer a level of protection in previously unvaccinated humans if given within four days after exposure. That window may be even wider with MVA. In either case, it appears that post-exposure vaccination could prevent or at least limit the severity of illness and thus will be indicated in people with smallpox exposure or even uncertain exposure to smallpox.

## 3. Vaccinia Immune Globulin (VIG)

Vaccinia immune globulin (VIG) is currently the only product that is approved by the FDA for treatment of complications from VACV vaccination and might have therapeutic efficacy in systemic orthopoxvirus infections caused by variola or monkeypox viruses. VIG is an isotonic sterile solution obtained by plasmaphoresis of people who were previously vaccinated with VACV and have high anti-VACV antibody titers [25]. In the past, VIG, administrated by the intramuscular route, was used to treat complications of smallpox vaccination. Due to limited stocks of the old preparations of VIG, in 2005, the FDA approved Dynport Vaccine Company LLC (USA) to manufacture new stocks of VIG. The new stocks of VIG can be administered intravenously (VIG-IV). In addition to being a less painful way to administer large volumes of VIG, intravenous administration seems to have more favorable pharmacokinetics than does VIG administrated intramuscularly [26]. When given intravenously, the geometric mean titer of VIG (given at a dose of 100 mg/kg) is 2.5-times higher than after intramuscular injection. Also, intravenous administration of higher doses (e.g., 200 mg/kg or 500 mg/kg), which would be difficult to give intramuscularly, is well tolerated. The older intramuscular VIG preparation contained the antimicrobial preservative thimerosol and was therefore contraindicated in pregnancy because of potentially teratogenic effects [27]. Although this specific risk does not exist with the newer intravenous preparations of VIG, VIG-IV is not recommended for use in pregnancy since it could potentially induce other adverse effects. However, it has been pointed out that antibody preparations used in pregnant women for the prevention of Rh incompatibility and treatment of idiopathic thrombocytopenic purpura are produced by the same processes as VIG-IV and have been safely administered to pregnant women [27].

The potency of VIG is defined by its ability to neutralize mature virus (MV, also called intracellular mature virus or IMV). However, during infection multiple forms of infectious virions are formed. The MV is a more stable form of the virus and is believed to be the form that is transmitted from host-to-host. The other important form of infectious virus is called the extracellular virus (EV, also called extracellular enveloped virus (EEV)), which is critical for spread of the virus from cell-to-cell and to distant sites within a host. Given the importance of EV in pathogenesis, activity of VIG against EV has also been examined. The major target of EV neutralizing antibody in VIG is against the EV-specific B5 protein [28,29]. VIG also contains antibodies that react with viral encoded immune evasion molecules (e.g., [30]), which could also play a role in its anti-poxvirus activity. Researchers have begun to try to identify all of the VACV targets in VIG [31,32]. Due to the highly homologous proteins encoded by orthopoxviruses, there is a high degree of shared serologic cross reactivity. Thus VIG could react with proteins from other orthopoxviruses and have similar therapeutic effects against other poxvirus infections.

VIG was first introduced into clinical use to treat the complications of smallpox vaccinations by Dr. Henry Kempe, a pediatrician practicing at the University of Colorado [33]. Kempe championed the use of VIG based on his astute clinical observations. He reasoned that antibodies could protect from VACV infections because he observed that the natural passive transfer of maternal anti-vaccinia antibodies to babies resulted in unsuccessful smallpox vaccination of some recently born babies [34]. He further believed that the development of the post-vaccination complication known as eczema vaccinatum was due to a delay in antibody production and that progressive vaccinia resulted from a complete failure to produce anti-vaccinia antibodies [33]. Thus, he was convinced that giving antibody as hyperimmune vaccinal gamma globulin (we will call this product VIG) could help patients who developed serious complications from smallpox vaccination. He modified this hypothesis in the late 1960s when he recognized that children with X-linked Bruton agammaglobulinemia could be safely vaccinated without vaccine related complications, but infants who had defects in delayed-type hypersensitivity reactions (now known as severe combined immunodeficiency) developed progressive vaccinia [35]. Thus, he believed that both cellular and humoral immune responses were important to control the infection [36,37]. Initially, Kempe studied the therapeutic use of VIG in a limited clinical trial in India to examine its usefulness after smallpox exposure [33]. In the 1960s, Kempe started to produce purified immunoglobulin and administered it to patients in the U.S. with serious vaccinia vaccination complications. Of note, no controlled clinical trials with VIG were ever done, but VIG became the accepted treatment based on the impression that treatment resulted in improvement.

### 3.1. Prevention and Treatment of Eczema Vaccinatum with VIG in Humans

Eczema vaccinatum is a rare, but potentially life-threatening illness that can occur in some people with atopic dermatitis or eczema that are exposed to VACV either by vaccination or by coming into contact with someone who has recently been vaccinated. Clearly not all people with these skin disorders develop eczema vaccinatum. The immune defects in the subset of patients at risk of developing eczema vaccinatum has not yet been fully defined, but animal models have implicated both innate and cellular responses (e.g., [38–40]). In 1956, Kempe *et al.* reviewed the prophylactic and therapeutic value of VIG to prevent and treat complications of smallpox vaccination [41]. They found that VIG protected children with skin conditions from developing complications of smallpox vaccination if VIG was given at the same time as smallpox vaccination. In this work, eight vaccinia-naïve children with eczema (three were <1-year old) required vaccination before going into an area where smallpox was endemic. The children received smallpox vaccination on normal skin and at the same time were given a single intramuscular injection of VIG. All the children developed the characteristic vesiculopustular lesion following primary vaccination, but none developed complications from vaccination. They also reported on the use of VIG in at risk children after direct vaccinia vaccination or after contact with someone recently vaccinated. The authors reported on six infants <18 months with eczema who were given VIG after a known accidental exposure to the vaccine. None of these children developed eczema vaccinatum [41]. The paper also reported on the treatment of eczema vaccinatum with VIG. Fourteen infants presenting with fairly severe eczema vaccinatum were treated with VIG once (or given a second dose later on). The first dose of VIG was given 2 to 5 days after presenting with eczema vaccinatum. In most cases, the children recovered, but there were two deaths that occurred on day 1 and day 3 after VIG administration. Both of the infants who died were already in a critical condition when VIG was started and the VIG was started five days after the onset of symptoms. A better outcome of eczema vaccinatum when treated early after presentation was illustrated in a recent report of this VACV vaccine related complication [42]. In this case, a two-year old child with a history of eczema and failure to thrive had a delay in treatment with VIG because the history of exposure to a parent who had been recently vaccinated with VACV was not immediately obtained. VIG treatment was begun ∼12 days after the potential onset of symptoms. Despite VIG treatment, the rash progressed and the child became critically ill requiring life support measures. At that point, in addition to VIG, the child was treated with cidofovir and an investigational new anti-poxvirus drug. Fortunately, the child ultimately recovered after a two-month hospitalization.

### 3.2. Use of VIG in Humans with Progressive Vaccinia and in Immunocompromised Animal Models

Kempe also used VIG to treat progressive vaccinia (at the time also known as vaccinia gangrenosum) and showed that some VIG treated children could survive this previously universally fatal disease [33,41,43]. In an excellent review on progressive vaccinia, Bray and Wright collected 41 reports of VIG treatment of progressive vaccina in children and adults from 1955 to 1997 [44]. Among 17 children with progressive vaccinia treated with VIG (± the antiviral drug methisazone), three (18%) survived. These three survivors had only partial immunodeficiencies like hypogammaglobulinemia. In the 14 children that died despite VIG treatment, seven had combined immune deficiencies, four had a cell-mediated deficiency and three had hypogammaglobulinemia, Among 24 adults with progressive vaccinia treated with VIG, 13 (54%) survived. Ten of these 13 survivors were individuals who developed immunodeficiency because of leukemia or lymphosarcoma treated with chemotherapy. Thus, VIG treatment of progressive vaccinia is more successful in adults than children, because adults with acquired immunodeficiencies were likely less immunosuppressed than the children. With that said, there is a case report of an adult with combined variable immunodeficiency that survived progressive vaccinia after treatment with VIG and methisazone [45].

Similar to the poor outcomes of immunocompromised children with progressive vaccinia treated with VIG, vaccinia infected mice with severe combined immune deficiency (SCID) treated with VIG also succumb to infection. In one study, the antiviral efficacy of VIG was studied in SCID mice infected with a recombinant VACV expressing the rabies virus glycoprotein [46]. SCID mice underwent intradermal scarification with 10^6^ pfu of the recombinant VACV on the dorsal surface of the tail with a bifurcated needle. Mice were then treated with an intramuscular dose of VIG (20 μL, which represented 1.2 mL/kg or ∼200 mg/kg of VIG) three times per week until day 34 post-infection. While during the first 14 days after infection, treated animals had significantly smaller tail lesions than untreated controls, both treated and untreated mice needed to be euthanized between day 25 and 34 because of progressive lesions or 20% weight loss. Interestingly, in this study the authors also combined VIG treatment with HPMPC (an acyclic nucleoside analog with anti-vaccinia activity) and showed that this group of treated mice did the best with smaller lesions, and no new skin lesions when therapy was stopped. All mice in this group survived until the experiment was terminated at day 65. The use of VIG along with an antiviral to treat poxvirus infections was a concept that Kempe also recognized as a potential way to treat progressive vaccinia [47].

In another study that looked at the biological activity of VIG-IV in SCID mice, Shearer *et al.* gave a single intravenous dose of VIG to SCID mice that were challenged with VACV [48]. In this study, the VACV challenge route was intravenous, but we are including it in this section on vaccine related infections because the challenge virus was the vaccine Dryvax (administered at a dose of 3 × 10^4^ pfu). Thus, this might represent a stage of viremia after vaccination in an immunocompromised host. They treated mice with a single dose of VIG (either 2.0 mg (100 mg/kg) or 8.0 mg (400 mg/kg)) the day prior to challenge, right after challenge (day 0), or on days 1, 2, 4, 6 or 8 after challenge. When given the day prior to challenge, both doses of VIG-IV prolonged survival. The 8 mg and 2 mg doses gave 80% and 40% survival, respectively, at day 49 (when the experiment was terminated). All untreated control mice succumbed to infection by about day 40. A somewhat similar result was found in mice treated immediately after intravenous challenge, with the 8 mg and 2 mg doses giving 80% and 20% survival, respectively. With each additional day after challenge when VIG was administered, the trends showed treatment to be less and less effective with decreasing survival rates and survival times in the treatment groups. By day 6 after challenge, the initiation of VIG treatment resulted in identical outcomes as the untreated controls. Thus, in hosts with severe combined immunodeficiency, VIG treatment can extend survival, but cannot cure the VACV infection.

### 3.3. Use of VIG in Humans after Exposure to Variola Virus

While the early use of VIG was examined to treat complications of VACV vaccination and after exposure to VARV, it is important to emphasize the differences in pathogenesis between a VACV skin lesion and the disseminated disease caused by VARV. During normal vaccination, the immune system must detect, control, and ultimately eliminate spread of virus at a single cutaneous site. T-cells responses (either CD8^+^ T cells or T helper cell-dependent antibody responses) are critical for control [49,50]. In smallpox, an inhaled dose of virus initially replicates in lymphoid tissues, then disseminates through the bloodstream. So the presence of protective antibodies through prior vaccination is a critical component in the protective immune response [22–24]. Thus the potential use of VIG after exposure to VARV has some rationale.

In 1953, Kempe examined the use of VIG in patients in India who had been exposed to family members with smallpox [33]. He studied 131 family contacts of 29 proven smallpox cases that were admitted to the Madras Infectious Disease Hospital. The contacts were alternately assigned to a group that received smallpox vaccination only or to a group that received vaccination and VIG. Among 75 contacts that received vaccination alone, there were eight (10.7%) secondary cases of smallpox. Five of these eight cases occurred in children under five years old and three of these children, who were all infants, died of smallpox. In the group of 56 contacts who received vaccination and VIG, there were only two (3.6%) secondary cases of smallpox. One of the smallpox cases was fatal and the other case developed mild disease. It is important to point out that both the vaccine only group and the vaccine plus VIG group had the same proportion of children under five years old. Kempe concluded that post-exposure smallpox vaccination along with VIG conferred better protection than vaccination alone [33]. These findings were replicated in a larger study [51]. In this study, among 326 contacts receiving VIG followed by vaccination, five (1.5%) cases of non-fatal smallpox developed while among 379 contacts that received vaccination alone there were 21 (5.5%) cases of non-fatal smallpox. Combined, these two studies showed ∼70% reduction in secondary smallpox in groups that received VIG and vaccination when compared to vaccination alone. Similar positive results with VIG were seen in a review [52] that collected a number of case reports of the use of VIG after prolonged exposure to smallpox. In these reports [53–55], all patients were vaccinated after exposure, but given the prolonged exposure times to the cases of smallpox, they were all felt to be beyond the window where therapeutic vaccination would be successful. Thus, 13 adults and 16 children were treated intramuscularly with VIG. All were protected except one adult and one child (both from the same report [54]) who developed very mild cases of smallpox. Similarly, in a report of 42 people who had close contact with cases of smallpox and some with delayed post-exposure vaccination, Marennikova showed a potential benefit of VIG [56]. None of the 13 contacts that were intramuscularly treated with VIG 5 to 22 days after exposure developed smallpox. Of note, 10 of these 13 received post-exposure vaccination five or more days after exposure. While none of these patients who received VIG developed smallpox, 13 (45%) of the 29 contacts that did not receive VIG developed smallpox. In this same report, Marennikova also reported the use of VIG to treat four patients with smallpox. Two of these patients (a 40 year old and an eight year old) were in the prodromal period and were given multiple doses of VIG on the third and fourth day of disease. They both had very mild disease with one being classified as smallpox without eruption. She also treated two patients with VIG “at the height of the disease” and observed that after treatment the patients clinically improved and lesions may have resolved more quickly [56]. Based on this human data, after a known smallpox exposure, VIG (along with vaccination) would be indicated. This will be particularly important in unvaccinated contacts. The use of VIG along with vaccination in people exposed to smallpox could help limit the spread and also lessen the mortality from an outbreak.

### 3.4. Use of VIG in Animal Models to Treat Orthopoxvirus Infections

The use of VIG for post-exposure treatment of poxvirus infections has been reexamined using various animal models. The therapeutic value of a rabbit vaccinia-immune IgG was investigated in mice intranasally challenged with VACV (strain WR) [57]. Law *et al.* found that giving hyperimmune anti-vaccinia rabbit antibody on day 3 post-infection was almost as protective as antibody given one day before infection. However, if the antibody was given four days after infection, poorer protection (as measured by weight loss) was seen [57]. Lustig *et al.* reported similar results with VIG in ECTV and VACV-WR challenge models [58]. If mice were intraperitoneally treated with a single dose of VIG (8 mg, 450–500 mg/kg) one day before or one day after challenge, full protection was obtained against intranasal challenge with lethal ECTV infection (5 × LD_50_). Even when VIG was given two or three days post-challenge, it conferred 60–80% protection. Interestingly, while VIG treatment given either one day prior or 1 day after intranasal challenge with VACV-WR (10^5^ pfu, ∼5 × LD50) resulted in 100% survival, both the pre and post-treatment groups had significant weight loss of >30%. This again shows that the differences in the pace of infection by the challenge viruses (and the subsequent ability of the host to mount a response to the infection) impacts on morbidity.

VIG has also been studied as treatment for systemic poxvirus infections in immunocompromised mice. The study by Lustig *et al.* also examined VIG treatment of SCID mice infected with VACV [58]. They either gave a single intraperitoneal injection of VIG (8 mg, 450–500 mg/kg) 24 hours prior to intranasal VACV-WR challenge (1.2 × 10^3^ pfu; ∼5 × LD_50_) or alternatively they administered 8 mg VIG prior to challenge followed by repeated injections of 2 mg VIG every other day for 18 days (thus a total VIG dose of 26 mg was given to the repeated dose group). As opposed to the untreated SCID mice, which all died by day 11 after challenge, the mice that received a single dose of VIG prior to challenge survived until day 16. The group of SCID mice that received repeated doses of VIG survived longer (100% at day 18, but 0% by day 22). Also in the group that received repeated VIG treatment, the mice had an early plateau in weight loss during the first week post-challenge, but then showed continuous weight loss until death. Again it seems that prolonged VIG treatment can extend survival, but adaptive immune responses are eventually needed for recovery.

These data in immunocompetent and immunocompromised animals suggest that high dose VIG treatment at times just prior or after a lethal orthopoxvirus challenge can alter disease. However, since these models use challenge doses and challenge routes that do not mimic natural acquisition that have a longer asymptomatic incubation period prior to the onset of disease, one potentially can look at this type of animal data as showing the likely effectiveness of antibody treatment if given during the incubation period. However, the benefit of antibody treatment greatly diminishes with the onset of symptoms.

### 3.5. Conclusions

In conclusion, VIG is the only product in the U.S. for treatment of complications of vaccinia vaccination. It gained approval at a time when specific antiviral drugs did not exist and antisera were the mainstay of infectious disease therapy. Once smallpox was eradicated, there was no motivation to develop antiviral therapies, so VIG remained the only licensed product. If smallpox had first appeared as a disease in the 1980s or later, it seems certain that the therapeutic approach would have focused on antiviral drugs and not on antibodies. Our current use of VIG therefore reflects its history, not its efficacy. While Kempe’s early observations that maternally transferred antibody to newborns prevented successful vaccination, VIG treatment does not prevent the formation of the post-vaccination vesiculopustular lesion. This finding allows successful VACV vaccination in the setting of concurrent VIG. But this finding also indicates that in the absence of other immune responses, VIG alone cannot fully control the resolution of a primary vaccination lesion. Currently there are two intravenous formulations of VIG (Dynport and Cangene) that have been issued a license by the FDA. Intravenous VIG formulations have advantages over the old intramuscular VIG preparations since it is easier to administer large volumes, distributed more rapidly, and contains no mercury derivatives. Experimental results in animal models support a role for VIG in prophylaxis and treatment of orthopoxvirus infections in animals with a functioning immune system. A more limited role is found in animals with severe combined immune deficiencies. Similarly in humans, VIG has a role in prophylaxis and treatment of eczema vaccinatum and in post-smallpox exposure prophylaxis (along with vaccination). Its use in immunocompromised hosts with progressive vaccinia has shown that it can alter what was a uniformly fatal complication from VACV vaccination. However, the efficacy in some patients with progressive vaccinia (and not others) is likely due to some patients having a partially functioning immune system. Thus, in order to enhance survival of patients where VIG has been ineffective, it will be important to improve immunoglobulin therapies. The current development of specific monoclonal antibodies to enhance VIG’s activity and/or specific anti-poxviral drugs that can be used with VIG might improve outcomes of patients with severe combined immunodeficiencies.

## 4. Anti-poxvirus Monoclonal Antibodies as Future Therapeutics

While VIG is believed to be effective in the treatment of early stages of eczema vaccinatum, its limited efficacy in treating progressive vaccinia and smallpox disease has lead researchers to pursue ways to augment the activity of anti-poxvirus immunotherapy. While some may think that in modern times, where careful screening of adults who require smallpox vaccination diminishes the need for the improvement of a VIG product, the continued rare, but serious adverse events after VACV vaccination show that screening cannot be 100% effective [42,59]. Furthermore, in the setting of the emergency use of smallpox vaccination in the outbreak setting, the reliance on screening may become even more difficult. Also, while the development of a more attenuated live VACV vaccine (e.g., MVA), may allow safer vaccination of people deemed at higher risk from complications from the conventional vaccine, here again in the emergency setting, where the conventional vaccine may be used, vaccine related complications could still arise. Thus, improving VIG is important in biodefense preparedness. Future approaches may be to include monoclonal antibodies (MAb) to increase VIG’s potency or to develop a defined cocktail of MAbs that may some day replace VIG. Development of a defined set of MAbs to replace VIG has the additional advantage of moving away from an immunoglobulin product that relies on human donors. This is not only important because of the ever present danger of human blood products containing an unscreened infectious agent, but the need for a non-human source product was highlighted in a recent report of the massive amounts of VIG used to treat a patient suffering from progressive vaccinia [59]. The patient was given a total of 16.7 million units of VIG. This amount of VIG was 30-times greater than the anticipated use of VIG for vaccine-related complications. This brings into question the amount of VIG in the national supply should widespread smallpox vaccination become necessary. There is some debate about the amount of VIG that was used to treat this specific patient, and whether VIG treatment was being used to control the lesion at the vaccination site, which as mentioned earlier may not be attainable with VIG alone. Nevertheless, to find alternatives to VIG, researchers have mainly focused on the generation of MAbs to the surface proteins on the two major forms of infectious VACV, mature virus (MV) and the extracellular virus (EV) [60–62]. Key neutralizing antibody targets on the MV envelope are encoded by the A27L [63], A28L [64], D8L [65], H3L [66], and L1R [67,68] genes. The A33R and B5R genes encode key antibody targets on the EV envelope [69].

One of the first identified targets of neutralizing monoclonal antibodies was the 14-kDa protein [63] encoded by the A27L gene. The VACV A27 protein is found on MV and is highly conserved among orthopoxviruses. Ramirez *et al.* used the anti-A27 mouse MAb C3 and studied its ability to protect Balb/c mice following a single administration either before and after VACV challenge [70]. Groups of mice were pretreated intraperitoneally with 10 or 100 μg of purified MAb C3 and then one hour later were intraperitoneally challenged with a lethal dose (1 × 10^8^ pfu) of a recombinant VACV expressing luciferase (WRluc). Mice pretreated with either dose of MAb C3 survived challenge, but untreated control mice died within three days after infection. Using whole mouse imaging, they also examined luciferase levels in target organs (ovaries and spleens) from mice that were first pre-treated with 1, 10, or 100 μg of MAb C3 and then intraperitoneally challenged one hour later with a lower dose (5 × 10^7^ pfu) of WRluc (so that mice would survive to day 5). When compared to untreated controls, luciferase levels were markedly reduced in organs of mice pretreated with 10 or 100 μg MAb C3. Since both the antibody and challenge virus were delivered intraperitoneally, the survival and decreased spread to organs could have been due to near complete neutralization of the challenge virus. Thus, they also examined post-challenge treatments. They intraperitoneally infected groups of mice with a sub-lethal dose of WRluc (2.5 × 10^7^ pfu) and measured virus spread in organs by whole mouse imaging. When compared to untreated mice, groups treated with 10 μg MAb C3 on days 1, 2, or 3 after challenges had decreased amount of virus in organs. These results showed that in a non-lethal intraperitoneal challenge model, an anti-A27 antibody could alter the spread of virus in the organs if given up to three days after infection. In addition to this, they also looked at survival of mice treated with MAb C3 (10 μg) one day after the higher dose (1 × 10^8^ pfu) lethal intraperitoneal challenge. While the mice pre-treated with MAb C3 were fully protected, the mice that were treated one day after challenge developed weight loss and limited survival. The poorer outcome of post-challenge treatment with antibodies to a MV target was confirmed in other studies that used an intranasal VACV challenge [71,72]. In these studies either mouse MAbs or rabbit polyclonal antibodies raised to another MV target (L1) conferred some protection if given prior to challenge, but provided poorer protection if given after challenge. As discussed next, these results support a model where a cocktail of antibodies to at least one MV target and one EV target would provide the best therapeutic effect [71].

While it makes sense that anti-MV antibodies help control the initial infecting MV inoculum, antibodies to EV are likely important in helping to control spread of an established infection. The protective role of individual anti-EV proteins were first shown by Galmiche *et al.* [69]. They tested rabbit antibodies raised to EV proteins A33, A34, A36, and B5 and found that subcutaneous administration of polyclonal rabbit antibody raised to A33 or B5 (10 μg/per injection, four-times at 2-week intervals; first dose given prior to challenge) protected mice against intranasal challenge with 107 pfu of VACV (strain IHD-J). Interestingly, while the anti-B5 antibodies had EV neutralizing activity in a plaque reduction assay, the anti-A33 antibodies were non-neutralizing. However, the anti-A33 antibodies (and anti-B5 antibodies) had anti-EV activity as measured by an EV-dependent spread assay called a comet inhibition assay.

Lustig *et al.* compared the protective effect of individual and combinations of mouse MAbs raised to A33, B5, and L1 [71]. They found that mice treated with a combination of anti-MV and anti-EV antibodies two days after challenge provided superior protection against lethal intranasal WR infection than treatment with antibodies raised to a single target. In these experiments, they treated mice with a combination of anti-A33 and anti-L1 MAbs (100 μg of each) after an intranasal lethal challenge with VACV-WR (1 × 106 pfu) and obtained 80 to 100% survival rates (with a maximum weight loss of ∼10 to 20%). However, treatment with a MAb raised to a single target gave 60–70% survival rates (with higher maximum weight loss of ∼25%). The study also examined treatment of infected SCID mice with the combination of antibodies. They intraperitoneally treated SCID mice with either a mixture of anti-A33, -B5, and -L1 polyclonal rabbit antibodies (1.67 mg of each) or with a mixture of anti-A33, -B5, and -L1 MAbs (100 μg each). Twenty-four hours after antibody treatment, they intranasally challenged the SCID mice with either 106 pfu (4000 × LD50) or 104 pfu (40 × LD_50_) of VACV WR. While all untreated mice challenged with 10^6^ pfu ultimately died, a one-time treatment with antibodies prior to challenge resulted in extended survival times when compared to the untreated controls. That is, while untreated controls all died within 11 days, mice treated with the rabbit polyclonals or MAbs had 50% survival times of 26 days and 46.5 days, respectively. Encouragingly, 40% of mice treated with the cocktail of MAbs and challenged with the lower WR dose (10^4^ pfu), survived more than three months (when the experiment ended), while untreated controls all died within two weeks.

Progress had been made in developing MAbs that would be suitable for human use. Chen *et al.* developed two chimpanzee/human anti-B5 MAbs that have high binding affinities to B5 [73]. *In vitro*, these antibodies could inhibit comet formation by VACV and VARV. *In vivo*, the MAbs provided significant protection against VACV challenge when given to mice two days after challenge. They found that intraperitoneal treatment with 90 μg of the chimpanzee/human anti-B5 MAb (8AH8AL) 48 hours after intranasal lethal challenge with VACV-WR (5 × 10^5^ pfu) protected mice from death (but they had weight loss of ∼15%). The group of mice that were not treated died within seven days after challenge. In these experiments they included a group of mice treated with a single dose of VIG (5 mg/mouse, ∼250 mg/kg) two days after challenge. VIG treatment afforded less protection than the chimpanzee/human anti-B5 MAb with a survival rate of 60%. Since the MAbs are therapeutic when administrated two days after exposure, the authors suggested that these MAbs may be a future alternative to VIG for treatment of complications from smallpox vaccination.

This group of investigators also isolated three anti-A33 chimpanzee/human antibodies, which had high binding affinities to A33 [74]. Similar to the anti-B5 MAbs, they showed that the anti-A33 MAbs reduced the spread of VACV *in vitro* by a comet inhibition assay. The MAbs also effectively protected mice from VACV infection, even when administrated two days after challenge. In these experiments, groups of mice were intranasally challenged with a lethal dose of VACV-WR (5 × 10^5^ pfu) and then 48 hours later the mice were treated with either 90 μg of a single MAb or a combination of an anti-A33 and anti-B5 MAb (45 μg of each MAb) or VIG (5 mg). All treatments protected mice from death. Again they found that post-challenge treatment with the anti-B5 MAb (8AH8AL) provided the best protection (with <5% weight loss). The group treated with the anti-A33 MAb (6C) had ∼16% weight loss (which was similar to the VIG treated group). The combination of anti-B5 and anti-A33 MAbs provided slightly better protection than anti-A33 alone with ∼7% weight loss). While the mouse experiments indicate that these anti-A33 MAbs are not as potent as the anti-B5 MAbs, the anti-A33 MAbs could have better activity in a non-human primate model.

While studies of anti-poxvirus antibodies have mainly focused on the direct neutralizing activity of the antibodies, the *in vivo* activity of therapeutic antibodies may also be enhanced by effector functions of the Fc domain on the antibody (e.g., [75]). For example, Benhnia *et al.* showed that the neutralization of EV by anti-B5 MAbs (with the proper Fc domain) is enhanced in the presence of complement [76]. These investigators also showed that because EV proteins are expressed on the surface of infected cells, the complement fixing MAbs could also promote complement mediated lysis of VACV infected cells. In mouse experiments they found that the level of protection conferred by passive transfer of an anti-B5 MAb is diminished if complement is transiently depleted. That is, in non-complement depleted mice first passively transferred with a single 100 μg dose of an anti-B5 mouse MAb, B126, survived intranasal challenge of VACV strain WR (5 × 10^4^ pfu; 1 to 3 × LD_50_) and showed no weight loss. In complement-depleted mice (by treatment of mice with cobra venom factor), the passively transferred MAb protected mice from death, but the mice had ∼15% weight loss after challenge. These data show the potential importance of complement for clearance of EV and VACV infected cells. Follow-up studies with human MAbs raised against B5 have confirmed that the Fc domain can alter the functional activity of the antibody in both complement dependent EV neutralization assays, as well as in direct complement-dependent cytotoxicity of VACV-infected cells [76,77].

Besides the approach of using therapeutic antibodies that target viral envelope proteins, another strategy for therapeutic antibodies would be to target viral proteins that are important for virulence. Orthopoxvirus, such as VARV, VACV, and ECTV, encode multiple immune response modifiers that are important for virulence [78]. One example of such a protein encoded by poxviruses is the type 1 interferon binding protein (IFN-bp). Xu *et al*. showed that it is essential for ECTV virulence and could be an effective target for protective immunization [79]. In this work, a recombinant ECTV in which the gene encoding the IFN-bp was deleted was >10^7^-fold attenuated in Balb/c mice infected by the footpad route with 3000 pfu of ECTV. The investigators also showed that the IFN-bp could be used as a subunit prophylactic vaccine to protect against subsequent ECTV challenge. This opens the possibility that antibodies raised to such immune response modifiers could be used in passive therapy of poxvirus infections.

In conclusion, MAbs generated to proteins on the two major forms of infectious VACV, mature virus (MV) and the extracellular virus (EV), have prophylactic and therapeutic effects against orthopoxvirus infections. Combinations of MAbs that recognize both MV and EV targets provided the best therapeutic effect. Replacing VIG, which requires human donors, with a defined cocktail of MAbs or the use of MAbs to enhance the activity of VIG, especially in immunocompromised hosts, is quite promising.

## 5. Conclusions

Given concerns about serious human orthopoxvirus infections that can be transmitted from person-to-person or are continuously introduced into human populations from a zoonotic source, it is important to have strategies for their efficient and immediate treatment. The conventional VACV-based live smallpox vaccine was an incredibly successful prophylactic vaccine that helped eradicate smallpox. Limited clinical studies also showed that if given early enough after exposure to VARV, the vaccine could modify and potentially prevent smallpox disease. More recent animal studies support the use of post-exposure vaccination, especially the mouse models that use ECTV challenge, which has a lag period before the onset of disease. It is also interesting that animal studies have revealed that when compared to conventional VACV vaccines, the MVA vaccine, which does not replicate in most mammalian cells, provides more rapid protection against lethal poxvirus. But this may be due to the higher doses used with MVA vaccination. Importantly, the animal studies have shown that the protection conferred by therapeutic vaccination is not simply through activation of innate immune responses, but that adaptive immunity must develop for protection. The use of VIG along with VACV vaccination also appears to be a useful strategy for those exposed to an active case of smallpox. Clinical work with VIG has shown it to have prophylactic and therapeutic value in preventing and treating vaccine-related complications like eczema vaccinatum. However, its efficacy to treat progressive vaccinia, especially in the patients with severe combined immunodeficiencies, is less encouraging. Animal studies of the therapeutic efficacy of VIG to treat systemic poxvirus infections in immunocompetent mice have demonstrated efficacy in post-exposure treatment of ECTV-challenged mice. The development of potent anti-poxvirus monoclonal antibodies, many that are now in a form suitable for human use, show great promise. They can be used to enhance the potency of VIG or to create a defined cocktail of antibodies, which also has the advantage of not relying on human donors.
